# Arthropod-borne diseases among travellers arriving in Europe from Africa, 2015 to 2019

**DOI:** 10.2807/1560-7917.ES.2023.28.7.2200270

**Published:** 2023-02-16

**Authors:** Céline M Gossner, Luisa Hallmaier-Wacker, Olivier Briet, Joana M Haussig, Henriette de Valk, Ariana Wijermans, Tamas Bakonyi, Theresa Madubuko, Christina Frank, Harold Noel, Mohammed Abdulaziz

**Affiliations:** 1 European Centre for Disease Prevention and Control, Stockholm, Sweden; 2 Santé publique France, Saint Maurice, France; 3 Africa Centres for Disease Control and Prevention, Addis Ababa, Ethiopia; 4 Robert Koch Institute, Berlin, Germany

**Keywords:** travel, malaria, dengue, chikungunya, surveillance, Africa

## Abstract

**Background:**

Travellers are generally considered good sentinels for infectious disease surveillance.

**Aim:**

To investigate whether health data from travellers arriving from Africa to Europe could provide evidence to support surveillance systems in Africa.

**Methods:**

We examined disease occurrence and estimated risk of infection among travellers arriving from Africa to Europe from 2015 to 2019 using surveillance data of arthropod-borne disease cases collected through The European Surveillance System (TESSy) and flight passenger volumes from the International Air Transport Association.

**Results:**

Malaria was the most common arthropod-borne disease reported among travellers from Africa, with 34,235 cases. The malaria travellers’ infection rate (TIR) was 28.8 cases per 100,000 travellers, which is 36 and 144 times higher than the TIR for dengue and chikungunya, respectively. The malaria TIR was highest among travellers arriving from Central and Western Africa. There were 956 and 161 diagnosed imported cases of dengue and chikungunya, respectively. The highest TIR was among travellers arriving from Central, Eastern and Western Africa for dengue and from Central Africa for chikungunya in this period. Limited numbers of cases of Zika virus disease, West Nile virus infection, Rift Valley fever and yellow fever were reported.

**Conclusions:**

Despite some limitations, travellers’ health data can efficiently complement local surveillance data in Africa, particularly when the country or region has a sub-optimal surveillance system. The sharing of anonymised traveller health data between regions/continents should be encouraged.

**Key public health message**

**What did you want to address in this study?**

Efforts to strengthen surveillance within Africa are ongoing. However, information about infections in some countries remains scarce. We wanted to examine the most common infections that European travellers acquire in Africa from vectors such as mosquitoes and understand how collection of such information could help improve surveillance in Africa.

**What have we learnt from this study?**

We learned that malaria was the most common disease among travellers from Africa to Europe, and is more frequent than dengue and chikungunya. Other mosquito-borne infections among European travellers were also reported but only sporadically. We confirmed that health data from European travellers can complement local surveillance data in Africa.

**What are the implications of your findings for public health?**

Our analysis can support travel advice and prevention policies with regards to vector-borne infections in travellers to Africa. It can also help to raise awareness among clinicians to consider these diseases in their diagnosis when treating returning travellers and to create awareness among travellers. Our analysis highlights areas in Africa that would benefit from increased surveillance.

## Introduction

Arthropod-borne diseases are diseases acquired through arthropod vectors, most often from the bite of infected mosquitoes, ticks, sandflies, or fleas. The burden of these diseases is predominantly carried by developing countries, including countries of the African continent.

In 2017, the Africa Centres for Disease Control and Prevention (Africa CDC) was established to support public health initiatives across the continent and to strengthen the capacity of its member countries to detect, prevent, control and respond to disease threats. In October 2020, the European Centre for Disease Prevention and Control (ECDC) and Africa CDC signed a 4-year partnership agreement aimed at strengthening preparedness and response, facilitating harmonised surveillance of outbreak-prone communicable diseases at the continental level and reinforcing the implementation of the Africa CDC’s public health workforce development strategy.

Despite the ongoing efforts to strengthen surveillance within Africa, information about pathogen circulation in some countries remains scarce. For countries with limited laboratory capacity, surveillance and reporting of infectious diseases, people travelling from these countries to countries with comparatively higher diagnostic and surveillance capacities are considered good sentinels for surveillance and thus can provide valuable data for early warning and monitoring of the epidemiological situation [1-4]. Through this study, the ECDC and Africa CDC aimed to jointly assess travellers’ health data collected in Europe to provide actionable information. The information obtained would inform clinicians and public health experts of the potential risks of infections in travellers during their stay in Africa and would also facilitate the ongoing capacity building within the Africa CDC.

## Methods

### Geographical setting

For this study, the terms Europe and European countries refer to the 27 European Union (EU) countries, plus Iceland, Liechtenstein, Norway and the United Kingdom (UK).

**Figure 1:**
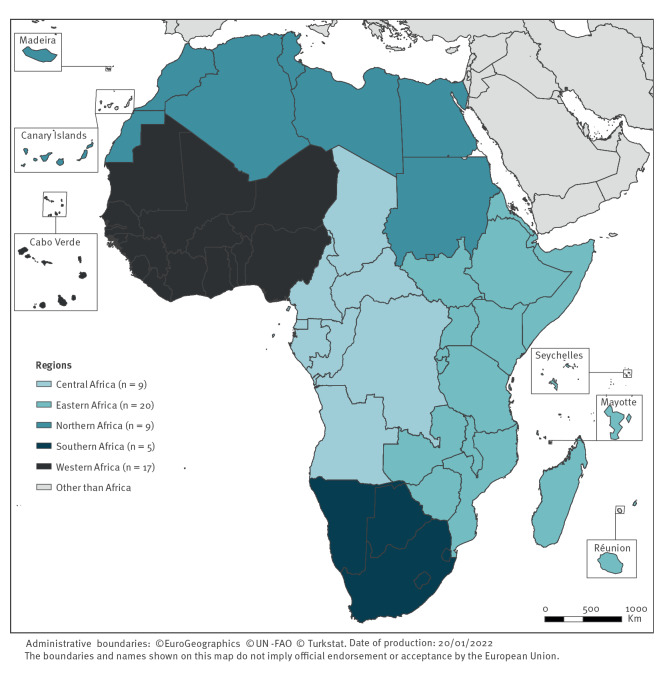
Regional grouping of African countries for the study, following the United Nations Statistics Division [5], 2015–2019 (n = 60 countries)

African countries were grouped into five regions following the United Nations Statistics Division [5] (Figure 1, see Supplementary Material S2 for the grouping of countries). The European outermost islands situated around the African continent were considered as countries geographically part of Africa.

The European outermost islands situated around the African continent were considered as countries geographically part of Africa.

### Travellers

International Air Transport Association (IATA) data were used to estimate passenger volume on commercial flights. The number of travellers from January 2015 through December 2019, with departure from an African country and arrival in a European country, were extracted on 30 July 2020. Direct and indirect flights (connecting flights) were used. Information on age and sex of travellers was not available in the IATA dataset.

### Disease cases

We used case-based data on mandatory notifiable arthropod-borne diseases at the European level extracted on 10 October 2021 from The European Surveillance System (TESSy) [6]. The following diseases were included: chikungunya, Crimean-Congo haemorrhagic fever (CCHF), dengue, malaria, plague, Rift Valley fever (RVF), tick-borne encephalitis (TBE), West Nile virus (WNV) infection, yellow fever and Zika virus disease (ZVD) [7].

We included probable and confirmed cases with a symptom onset date from January 2015 through December 2019. Classification (probable vs confirmed) was based on the EU case definitions [7]; case classification status was available for all diseases except ZVD. Laboratory diagnostic methods for disease confirmation are described in the EU case definitions. When symptom onset date was unavailable, we used the diagnosis date as a proxy or alternatively the date of notification to public health authorities, and ultimately the ‘statistics date’. The latter is the only mandatory date field and may refer to any of the dates mentioned above. Cases with the probable country of infection in Africa were selected. When several countries were mentioned as a probable country of infection, the case was excluded (n = 37 cases for malaria, n = 9 cases for dengue and n = 6 cases for chikungunya).

Detailed information about the inclusion criteria is provided in the Supplementary Material S1.

### Data analysis

We performed a descriptive analysis of the travellers’ volume data and the arthropod-borne disease cases.

For diseases with at least 100 cases per year (arbitrary cut-off), we calculated the disease-specific travellers’ infection rate (TIR), which we considered as a proxy for the likelihood of infection. TIRs were calculated for malaria, dengue and chikungunya as follows:


$$TIR=\frac{Number\;of\;cases\;reported\;in\;Europe\;and\;infected\;in\;Africa\;during\;a\;period\;t}{Number\;of\;travellers\;arriving\;in\;reporting\;countries\;in\;Europe\;that\;departed\;from\;Africa\;during\;a\;period\;t}*100,000$$

The 95% confidence intervals around the TIR followed a Poisson distribution. To limit bias linked to irregular and incomplete reporting, to errors in gathering or reporting of travel history/exposure of the cases or to the lack of specificity of IgM serology testing for dengue and chikungunya [8,9], we applied the following selection criteria to all three diseases: (i) we included disease cases reported by European countries that reported case numbers every year (including zero cases) during the studied period and provided the place of infection for at least half of their cases (arbitrary cut-off). Accordingly, only the travellers arriving from Africa to these European countries were included; (ii) we included countries of infections associated with at least two cases, of which one or more was a confirmed case, and that were either reported by two different reporting countries or reported over multiple years.

For the calculation of the regional TIR, we consistently included travellers departing from all the countries included in the defined region regardless of disease occurrence in all or some of the countries in the region. Detailed information about the inclusion criteria is provided in the Supplementary Material S1.

We used Stata software release 14 (StataCorp. LP) for data management and analyses. ECDC Map Maker tool (EMMa) was used to create maps.

## Results

### Travellers arriving from Africa

**Figure 2:**
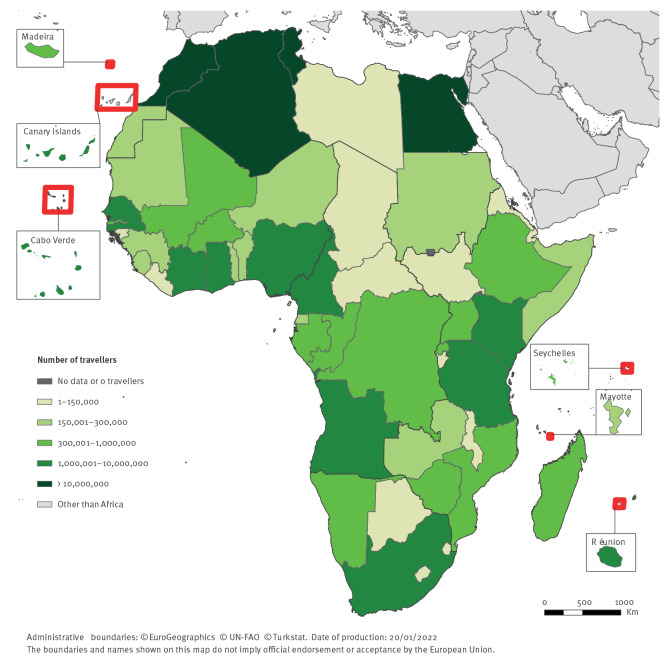
Number of travellers arriving in Europe from Africa, per country of departure, 2015–2019 (n = 125,050,069)

Overall, ca 125 million people arrived by commercial airplane from Africa to Europe from 2015 through 2019. The highest volume of travellers was observed from Northern Africa (79.3 million), mainly from Morocco (31.1 million) (Figure 2). There were 16.4 and 15.4 million travellers from Eastern and Western Africa, respectively, and there were 9.6 and 4.2 million travellers from Southern Africa (mostly from South Africa) and Central Africa, respectively.

The European outermost islands situated around the African continent were considered as countries geographically part of Africa.

The yearly number of travellers arriving from Africa to Europe increased consistently from 23.1 million in 2015 to 28.8 million in 2019, with the exception of 2016 when there was a decrease in number of arriving travellers compared to the previous year. The overall increase was more pronounced for Eastern and Western Africa, with a 36% and a 34% increase in 2019 compared to 2015, respectively. Travellers’ volume for Central Africa was 9% lower in 2019 compared to 2015.

### Imported cases and travellers’ infection rates

From 2015 through 2019, European countries reported cases of malaria (n = 34,235), dengue (n = 956), chikungunya (n = 161), ZVD (n = 16), WNV infection (n = 9), RVF (n = 4), and yellow fever (n = 1) imported from Africa, but no cases of CCHF, plague or TBE (Table 1). The annual case number and TIR per country, region and for Africa overall for malaria, dengue and chikungunya are provided in the Supplementary Material S2.

**Table 1:** Descriptive analysis of data on travellers from Africa to Europe and data on imported disease cases, per disease, 2015–2019 (n = 125,050,069 travellers)

Characteristics	Unadjusted number of travellers^a^	Malaria		Dengue		Chikungunya		Zika virus disease	West Nile virus infection	Rift Valley fever	Yellow fever
		n	TIR^b^	n	TIR^b^	n	TIR^b^	n	n	n	n
Overall	125,050,069	34,235	28.8	956	0.8	161	0.1	16	9	4	1
Classification											
Probable	NA	19	NA	114	NA	64	NA	1	0	1	0
Confirmed		34,216		842		97		8	9	3	1
Unknown or unspecified		0		0		0		7^c^	0	0	0
Region of infection											
Eastern Africa	16,381,969	3,877	24.3	505	3.2	84	0.6	1	1	0	0
Central Africa	4,278,672	9,151	225.6	92	2.3	48	1.2	8	0	0	0
Northern Africa	79,324,402	451	0.6	30	0.0	3	0.0	0	7	0	0
Southern Africa	9,618,811	99	1.1	18	0.2	0	0.0	0	1	0	0
Western Africa	15,446,215	20,657	140.0	311	2.1	26	0.2	7	0	4	1
Sex											
Male	NR	22,403	NA	547	NA	66	NA	8	5	4	1
Female		11,772		407		95		8	4	0	0
Unknown or unspecified		60		2		0		0	0	0	0
Age groups (years)											
Mean age (range)	NR	37 (0−93)	NA	42 (2–84)	NA	47 (18–88)	NA	42 (23–70)	65 (45–81)	32 (28–37)	26 (NA)
0–4		1,039		4		0		0	0	0	0
5–14		2,565		22		0		0	0	0	0
15–24		4,346		83		7		1	0	0	0
25–44		14,519		423		61		8	0	4	1
45–64		10,090		341		73		6	5	0	0
≥ 65		1,542		81		20		1	4	0	0
Unknown or unspecified		134		2		0		0	0	0	0
Year of disease onset											
2015	23,123,293	6,733	30.6	70	0.3	13	0.1	0	0	1	0
2016	21,782,484	6,445	31.1	128	0.6	35	0.2	4	2	3	0
2017	24,268,380	6,911	29.9	239	1.0	16	0.1	4	1	0	0
2018	27,112,632	6,966	27.0	146	0.6	40	0.2	1	4	0	1
2019	28,763,280	7,180	26.3	374	1.4	57	0.2	7	2	0	0
Outcome											
Alive	NA	15,966	NA	354	NA	41	NA	4	5	4	1
Dead		133		0		0		0	1	0	0
Unknown or unspecified		18,136		602		120		12	3	0	0
Place of residence											
Africa	NR	2,209	NA	8	NA	1	NA	0	0	0	0
- Eastern Africa		66		2		0		0	0	0	0
- Central Africa		1,025		4		0		0	0	0	0
- Northern Africa		32		0		1		0	0	0	0
- Southern Africa		10		0		0		0	0	0	0
- Western Africa		1,076		2		0		0	0	0	0
Europe		17,254		337		44		15	5	4	0
Americas		20		0		0		0	0	0	0
Asia		33		0		0		0	0	0	0
Oceania		6		0		0		0	0	0	0
Unknown		14,713		611		116		1	4	0	1

NR: data not reported; NA: not applicable; TIR: Travellers’ infection rate.

^a^ Total number of travellers to Europe.

^b^ Number of cases per 100,000 travellers; the number of travellers was adjusted to the reporting countries included.

^c^ EU countries did not specify how the cases were laboratory-confirmed and therefore the category into which these fall is unknown.

#### Malaria

Malaria was the most common arthropod-borne disease among travellers from Africa, with 34,235 cases (TIR = 28.8/100,000 travellers) (Table 1). Most of these were confirmed cases (> 99%). The number of cases consistently increased from 2015 to 2019, with the exception of 2016 when there was a decrease in number of malaria cases compared to the previous year; in 2019 the number of malaria cases was 7% higher than in 2015. The malaria TIR followed an opposite yearly pattern, the TIR was 14% lower in 2019 compared to 2015. Malaria-infected travellers arrived from 50 African countries, predominantly from Western Africa (n = 20,657; 60%) and Central Africa (n = 9,151; 27%). TIRs were highest for Central Africa (TIR = 225.6) and Western Africa (TIR = 140.0). 

**Figure 3:**
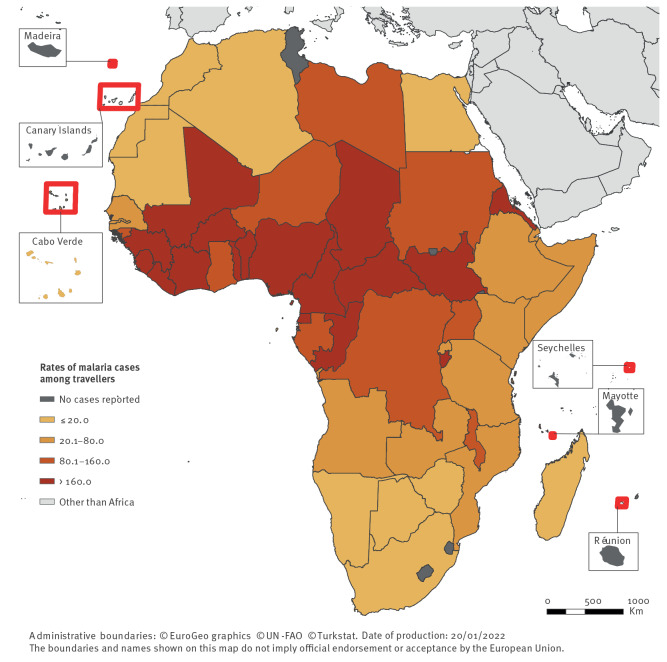
Rates of malaria cases per 100,000 travellers arriving in Europe from Africa, per country of infection, 2015–2019 (n = 34,235 cases)

For Western Africa, about half of the malaria cases were infected either in Nigeria (n = 5,822; TIR = 202.7) or in Côte d’Ivoire (n = 4,550; TIR = 404.7) (Figure 3 and Supplementary Material S2). For Central Africa, 44% of the cases were infected in Cameroon (n = 4,056; TIR = 425.9). TIRs were highest for the Central African Republic (n = 790; TIR = 1,608.5) and Sierra Leone (n = 1,071; TIR = 712.2).

The European outermost islands situated around the African continent were considered as countries geographically part of Africa. Rates of malaria cases are expressed per 100,000 travellers.

For some countries, the annual case number and associated TIR fluctuated; there were 210 cases from the Central African Republic in 2015 (TIR = 3,188.1) and 148 cases in 2019 (TIR = 1,159.9). Similarly, there were 129 malaria cases from Sudan in 2015 (TIR = 343.0) and 47 cases in 2017 (TIR = 73.8). The yearly variation in case number is not directly proportional to the yearly variation in TIR.

The *Plasmodium* species was specified for 92% (n = 31,404) of the cases. *Plasmodium falciparum* accounted for the majority of those cases (n = 28,070; 89%); the proportion of *P. falciparum* ranged from 75% for Eastern Africa to 92% for Western Africa. *Plasmodium ovale*, *P. malariae* and *P. vivax* represented 6%, 3% and 2% of the infections, respectively. The remaining infections (< 1%) were mixed infections with various *Plasmodium* species. Infections by *P. falciparum*, *P. ovale* and *P. malariae* primarily originated from Western Africa; infections by *P. vivax* primarily originated from Eastern Africa. The number of people infected with *P. vivax* in Eastern Africa decreased by 83% from 2015 to 2019, from 198 cases to 33 cases. In parallel, the *P. falciparum* infections in this region increased by 74%, from 377 cases in 2015 to 657 cases in 2019. While 31% and 59% of the malaria infections in Eastern Africa in 2015 were due to *P. vivax* and *P. falciparum*, respectively, these proportions were 4% and 87% in 2019. For Northern Africa, with 93% of cases originating from Sudan, a comparable shift in *Plasmodium* species distribution was observed with an increased proportion of *P. falciparum* cases in 2018 and 2019 compared to previous years, and a decrease in *P. vivax* those last two years compared to previous years. The proportions and numbers of malaria cases per *Plasmodium* species, year and region is presented in the Supplementary Material S3.

Sixty-five percent of the cases were male and the mean age at infection was 37 years. Among the cases that specified outcome (47%) the case fatality ratio was < 1%. Among the cases for which country of residence was specified (57%), 88% were European residents and 11% African residents, principally from Western Africa and Central Africa (Table 1).

#### Dengue

Dengue was the second most common arthropod-borne disease reported with 956 imported diagnosed cases (TIR = 0.8) (Table 1). The majority of the cases (88%) were confirmed. Two peaks in cases and TIR were observed, in 2017 (n = 239; TIR = 1.0) and in 2019 (n = 374; TIR = 1.4).

Infected travellers arrived from 41 African countries, predominantly Eastern (n = 505; 53%; TIR = 3.2) and Western Africa (n = 311; 33%; TIR = 2.1). Despite low case numbers from Central Africa, the TIR was comparable to that of Western Africa.

**Figure 4:**
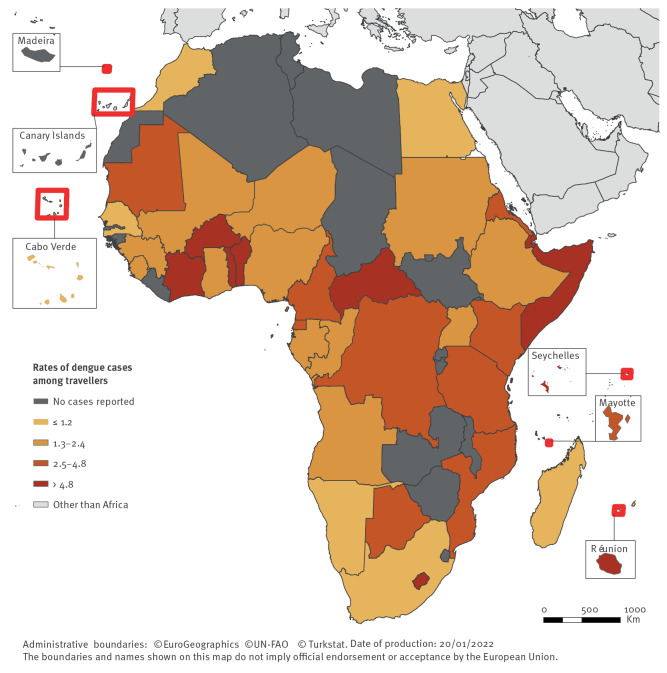
Rates of dengue cases per 100,000 travellers arriving in Europe from Africa, per country of infection, 2015–2019 (n = 956 cases)

Most cases from Eastern Africa were infected in Réunion (31%; TIR = 5.2) and most cases from Western Africa were infected in Côte d’Ivoire (36%; TIR = 9.9) (Figure 4 and Supplementary Material S2). The highest TIR was for travellers infected in Lesotho (TIR = 107.9) but the TIR confidence interval was broad, hence minimising the validity of the result. The second and third highest TIR were for travellers infected in Somalia (TIR = 16.6) and Burkina Faso (TIR = 14.8).

The European outermost islands situated around the African continent were considered as countries geographically part of Africa. Rates of dengue cases are expressed per 100,000 travellers.

Temporary prominent peaks in case numbers and TIRs were observed for Burkina Faso in 2016 and 2019 (TIR = 25.1 and 21.6), Côte d’Ivoire in 2017 and 2019 (TIR = 24.9 and 18.6), Réunion in 2019 (TIR = 18.1) and Seychelles in 2016 and 2017 (TIR = 11.3 and 21.8), among others.

No deaths associated with dengue were reported. Fifty-seven percent of the cases were male and the mean age at infection was 42 years. Among the cases with a country of residence reported (36%), 98% were European and 2% African residents.

#### Chikungunya

Between 2015 and 2019, there were 161 cases (TIR = 0.1) of chikungunya (59% confirmed) (Table 1). The annual case number and TIR were highest in 2016 (n = 35; TIR = 0.2), 2018 (n = 40; TIR = 0.2) and 2019 (n = 57; TIR = 0.2).

Infected travellers arrived from 20 African countries, predominantly from Eastern (n = 84; 52%), Central (n = 48; 30%) and Western Africa (n = 26; 16%). The TIR was highest among travellers arriving from Central Africa (TIR = 1.2).

**Figure 5:**
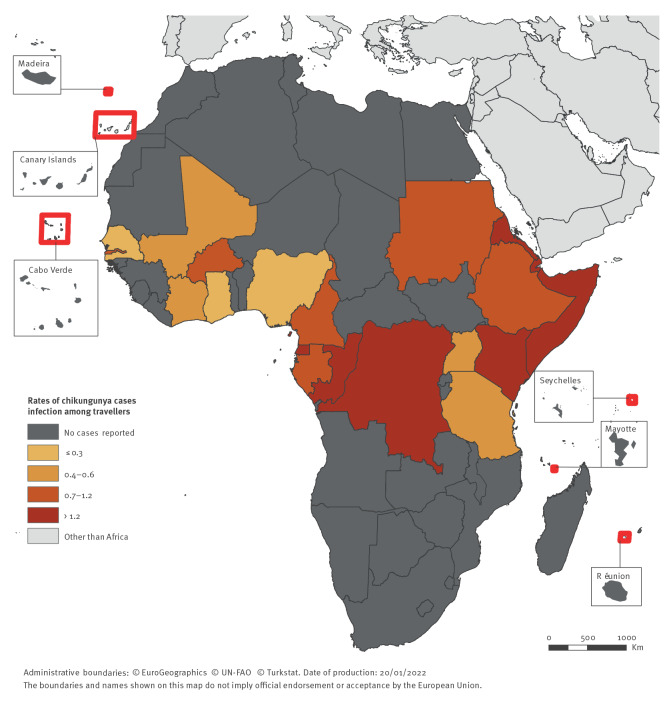
Rates of chikungunya cases per 100,000 travellers arriving in Europe from Africa, per country of infection, 2015–2019 (n = 161 cases)

Most cases arriving from Eastern Africa were infected in Kenya (40%; TIR = 1.4) or Somalia (35%; TIR = 15.0) (Figure 5 and Supplementary Material S2). In Central Africa, the main countries of infection were Congo (Brazzaville) (35%; TIR = 4.0), the Democratic Republic of the Congo (DRC) (25%; TIR = 3.8) and Equatorial Guinea (21%; TIR = 5.9). In Western Africa, Côte d’Ivoire (19%; TIR = 0.4), Nigeria (19%; TIR = 0.2) and Senegal (19%; TIR = 0.2) were the countries with the highest case numbers. The highest TIR was for travellers infected in Somalia, followed by travellers infected in Equatorial Guinea and Congo (Brazzaville).

The European outermost islands situated around the African continent were considered as countries geographically part of Africa. Rates of chikungunya cases are expressed per 100,000 travellers.

The most prominent peaks in case numbers and TIRs were observed in Somalia in 2016 (TIR = 65.0), Kenya in 2018 (TIR = 5.1), Congo (Brazzaville) and DRC in 2019 (TIR = 22.6 and 15.6, respectively).

No chikungunya associated deaths were reported. Forty-one percent of the cases were male and the mean age at infection was 47 years. Among the cases with a country of residence reported (28%; 45/161), 98% (n = 44) were European and 2% (n = 1) African residents.

#### Zika virus disease

There were 16 ZVD cases reported in 2016 (n = 4 cases), 2017 (n = 4), 2018 (n = 1) and 2019 (n = 7). These cases were infected in Angola (n = 4), Cameroon (n = 4), Burkina Faso (n = 2), Cabo Verde (n = 2), Côte d’Ivoire (n = 1), Kenya (n = 1), Nigeria (n = 1) and Senegal (n = 1). Half of the cases (n = 8) were women and the mean age at infection was 42 years. No ZVD cases among pregnant women or deaths were reported. Place of residence was reported for 15 cases; all were European residents.

#### West Nile virus infections

There were nine imported confirmed cases of WNV infection: five from Tunisia (1 in 2016, 3 in 2018 and 1 in 2019) and one each from Algeria (2018), Djibouti (2019), Egypt (2016) and South Africa (2017). Five of the cases were male and the mean age at infection was 65 years. One fatal case was reported. Place of residence was reported for five cases and all were European residents.

#### Rift Valley fever

There were four imported cases of RVF, all from Western Africa: three from Mali (1 confirmed case in 2015 and 2 confirmed cases in 2016) and one from Ghana (1 probable case in 2016). All cases were male and the mean age at infection was 32 years. All four cases were European residents. No associated deaths were reported.

#### Yellow fever

In 2018, one confirmed case of yellow fever was reported with exposure in Senegal. The case was a 26-year-old male who survived the infection. The place of residence of this case was unknown.

## Discussion

Malaria was by far the most frequently diagnosed arthropod-borne disease among travellers arriving from Africa to Europe, despite the existence of chemoprophylaxis. The malaria TIR was 36 and 144 times higher than the TIR for dengue and chikungunya, respectively. This reflects the high level of endemicity of the disease and transmissibility of the parasites in a large part of the African continent, the long durations of detectable (untreated) infections (as compared with the arboviral diseases) and the high proportion of cases presenting clinical manifestation making diagnosis likely. Geographically, the variation in TIRs over the regions correlates well with disease incidence estimated by the World Health Organization (WHO): low in Northern and Southern Africa, intermediate in Eastern Africa, and high in Central and Western Africa [10]. The number of imported cases to Europe has been slowly increasing but the likelihood of infection within Africa has decreased; this is likely the result of large efforts by African countries to limit the circulation of the parasites and also possibly of an improving adherence to prophylaxis over the years. The World Malaria Report 2020 estimates a 3.4% decline in case incidence in the WHO African Region over the 2015–19 period [10]. Several hypothesis could explain the faster decline of malaria incidence in travellers: (i) malaria incidence saturates at higher levels of endemicity in residents because of superinfection and acquired immunity, while malaria episodes in (non-immune and briefly exposed) travellers more closely reflect the force of infection, (ii) there might have been an increase in the use of prophylaxis among travellers during the study period and (iii) tourist travellers are thought to primarily visit regions that are more developed, in which there might be more and more effective malaria control activities.

Individuals were primarily infected by *Plasmodium vivax* in Eastern Africa, which aligns well with the existing epidemiological knowledge on the disease. However, there is a striking difference in the proportion of infections because of *P. falciparum* among imported malaria cases (72–90%, depending on the region) and those reported in the World Malaria Report 2020 (97–100%), the latter source having much lower estimates for the proportions of other species and mixed infections. Mixed infections are often underdiagnosed [11] and there may be a bias towards *P. falciparum* in settings that use rapid diagnostic tests that only detect this species [12]. Accurate species identification may have important consequences for the choice of treatment, as *P. vivax* and *P. ovale* can develop dormant liver stages (hypnozoites) that require specific drugs to avoid disease relapses. The decrease in the proportion of *P. vivax* in Eastern Africa in travellers (from 33% in 2015 to 4% in 2019), is reflected in estimates by WHO (from 3.1% in 2015 to 0.4% in 2019) [10].

Malaria was the disease with the highest proportion of cases in the African resident category (15%). This is likely due to malaria being the only arthropod-borne infection included in this study for which there is chemoprophylaxis, combined with an increased likelihood that European residents would take chemoprophylaxis compared with African residents.

The number of imported cases and TIRs were higher for dengue than for chikungunya. This may reflect a wider circulation of dengue virus compared with chikungunya virus especially in touristic areas. The endemo-epidemic patterns of dengue and chikungunya resulted in increases in numbers of cases and TIRs among travellers during epidemic years. Travellers seemed more likely to be affected when outbreaks occurred in the capital cities or when travelling to countries with more touristic areas, e.g. Ouagadougou in 2016–17 [13], Abidjan in 2017 and 2019 [14,15], Seychelles in 2016–17 [14] and Réunion in 2019 [16]. Outbreaks in non-touristic areas are less likely to be detected through analysis of traveller’s heath data. For instance, there were no clear signs of the dengue outbreak that affected the Louga region, Senegal, in 2017 and the chikungunya outbreak that affected the city of Dire Dawa, Ethiopia, in 2019 [14,17].

All but one ZVD case were imported from countries with known Zika virus circulation in the corresponding year [18]. The exception was a case from Kenya, a country not considered at risk by national [19] or international organisations (e.g. World Health Organization) [18] during the study period. Based on serological evidence indicating virus circulation [20-22], in 2022 the World Health Organisation added Kenya to the list of at-risk countries [23]. The maintenance of a list of countries at risk is paramount as, to date, pregnant women are still advised to avoid travel to those countries [24]. The peak of the ZVD pandemic was in 2016, but surprisingly, the highest number of imported cases from Africa was observed in 2019. In 2019 the first, and so far unique, autochthonous outbreak of ZVD was reported in mainland Europe [25]. This highlights that despite the large decrease in incidence worldwide, the virus remains a public health threat in Africa and in Europe [26].

Most cases of WNV infections were imported from Northern Africa, a region with previous evidence of virus circulation [27,28]. Considering that the vast majority of WNV infections remain pauci- or asymptomatic, the volume of travellers needs to be sufficiently high for WNV detection by sentinel surveillance. On average, individuals diagnosed with WNV were older than for other arthropod-borne diseases, which is concordant with the characteristic of the disease (i.e. affecting more severely elderly persons) and current diagnostic practices (i.e. more severe cases are more likely to be tested and diagnosed [29]).

The two cases of RVF reported in 2016 from Mali were members of the French armed forces [30]. Infections among European military personnel deployed in Africa have been previously reported, for instance in Chad and Egypt [31,32] and members of the military are considered at increased risk of infection. The case infected in Mali in 2015 was an immunocompromised individual who handled livestock and consumed raw cow milk [33]. There was one probable case imported from Ghana; no previous report of human or animal RVF infection in Ghana could be found in the literature, but a disease suitability model highlighted the risk for transmission in the country [34].

According to the literature, the imported case of yellow fever reported through TESSy was unvaccinated and travelled both to Gambia and to Senegal [35]. While there is yellow fever virus transmission in these two countries [36], there is no vaccination requirement for travellers at entry [37]. In contrast, there was a large outbreak of yellow fever in Angola and DRC in 2016–17 [38]; both countries require proof of yellow fever vaccination at entry. No related yellow fever cases imported to Europe were reported during these large outbreaks. Public health authorities and travel clinics should continue emphasising the importance of vaccination for travellers, even when visiting at-risk countries where vaccination is not legally required.

For CCHF, TBE and plague, this study found no cases in travellers from Africa in the period analysed. For TBE, there has been no evidence of human infections in Africa [39]. For CCHF, there are some known endemic regions and sporadic outbreaks have affected humans (e.g. four CCHF cases is Uganda in 2018 [40]). During the study period, outbreaks of plague in Africa have only been reported in Madagascar and, despite the large outbreak in Madagascar in 2017 with over 230 cases, no travel-related cases have been reported in Europe [41]. The rural and inland locations of the seasonal plague outbreaks, where the tourist volume is lower, may have contributed to a very low likelihood of infection in travellers from Europe [42].

The number of imported cases detected is influenced by at least four factors: (i) presence of the pathogen in the visited country and its intensity of circulation, (ii) exposure of travellers to pathogens and their susceptibility to infection, (iii) volume of people potentially exposed arriving to Europe (i.e. European travellers returning to Europe, African travellers visiting Europe or migrants) and (iv) sensitivity of the surveillance system in Europe. It is important to note that the case numbers and thus the TIRs presented in this study are likely an underestimation as not all cases are diagnosed and reported within Europe (e.g. cases who recovered before their arrival to Europe), and some infections remain pauci- or asymptomatic and thus not diagnosed. The more severe the disease, the more likely it is to be diagnosed. Conversely, not all travellers are moving by plane and migrants, who largely do travel by land and sea, would not be accounted for in the denominator when calculating of the TIR. This most likely led to a minor overestimation of the TIR, particularly for malaria. The TIRs presented in this study, however, help assess the risk of infection for the ’average’ traveller and highlight the need for continually educating travellers on arthropod-borne diseases and their preventative measures. The absence of information on length of stay in the country of exposure, category of traveller (e.g. tourists, visiting friends or relatives, migrants) and use of malaria chemoprophylaxis limited possible sub-analyses.

Collecting travellers’ health data is essential to assess the risk for travellers. However, for African countries, the usefulness of European surveillance data could be questioned. Travellers’ health data cannot replace local surveillance in providing accurate estimations of outbreak magnitude and regional incidence because (i) the number of travellers remain limited compared with the local population, (ii) both populations have different immunological competences to pathogens circulating in Africa, (iii) the use of prophylaxis medications differs and (iv) there are behavioural and habitual differences between the two populations. However, travellers’ health data can efficiently complement local surveillance data, particularly when the country or region has a sub-optimal surveillance system. Similarly, travellers might be index cases of yet unrecognised outbreaks, as exemplified by an American healthcare worker who was airlifted from Togo to Germany and diagnosed with Lassa fever in 2016, at a time when there had been no evidence of ongoing circulation of the virus in Togo [43]. When there is evidence of pathogen transmission in travellers from countries with no reported cases among the local population, it is important to enhance surveillance in the local population. For example, the report of imported dengue cases in this study among travellers arriving from Ghana (Supplementary Material S2) confirmed the suspicion of virus circulation since at least 2015 [44] and highlights the need for capacity building to ascertain the local incidence. As such, the European dataset can help identify areas in Africa for epidemiological investigations. It is, however, important to note that the absence of travel-related cases does not provide evidence for the absence of pathogen circulation and should thus not be used as a basis for relaxing public health measures in a region.

The strengths of TESSy data are its comprehensiveness and its representativeness, as reporting is mandatory and covers all EU/EEA countries. However, for most arthropod-borne diseases, the data are reported annually, which can support patterns and trend analysis but does not allow the early detection of outbreaks. For early detection of outbreaks among travellers, international sentinel surveillance networks such as EuroTravNet and GeoSentinel play a prominent role [4,45]. In addition, since 2021 public health institutes in EU/EEA countries are encouraged to report unusual events among travellers to ECDC and other public health institutes through EpiPulse, which is an event-based surveillance platform aiming at the timely detection and assessment of potential cross-border health threats to European citizens [46].

We deliberately did not include 2020 and 2021 data to avoid bias related to the coronavirus disease (COVID-19) pandemic. During these 2 years, the number of imported cases of arthropod-borne diseases from Africa drastically decreased because of the travel restrictions [47]. Despite uncertainty, we assume at this stage that the results presented in this study will be applicable after the COVID-19 pandemic.

## Conclusion

Malaria was by far the most reported arthropod-borne diseases among travellers arriving in Europe from Africa. Continued efforts are required to convey pre-travel advice to travellers to Africa and particularly to those most likely to be exposed to *Plasmodium* species. This work provides avenues for further collaboration between ECDC and the Africa CDC with the aim to strengthen surveillance in Africa and prevent the occurrence of arthropod-borne infections among travellers but also locally. The ability of the Africa CDC to conduct surveillance of health threats in Africa can be strengthened by having access to data from travellers that can complement locally sourced data. Overall, the sharing of anonymised traveller health data in accordance with data protection legislations between regions/continents should be encouraged.

## Supplementary Data

327000 Supplement
