# Supplementary material for: Arthropod-borne diseases among travellers arriving in Europe from Africa, 2015 to 2019
Source: Euro Surveill. 2023 Feb 16;28(7):2200270. doi: 10.2807/1560-7917.ES.2023.28.7.2200270 (PMC9936595; doi:10.2807/1560-7917.ES.2023.28.7.2200270)
Supplement: Supplement [file 22-00270_GOSSNER_SUPPLEMENT.pdf]

Arthropod-borne diseases among travellers arriving in Europe from Africa, 2015–2019

This supplementary material is hosted by *Eurosurveillance* as supporting information alongside the article Arthropod-borne diseases among travellers arriving in Europe from Africa, 2015–2019, on behalf of the authors, who remain responsible for the accuracy and appropriateness of the content. The same standards for ethics, copyright, attributions and permissions as for the article apply. Supplements are not edited by *Eurosurveillance* and the journal is not responsible for the maintenance of any links or email addresses provided therein.

Supplementary material 1

Study inclusion criteria per disease

Malaria

Probable and confirmed travel-related cases with (estimated) date of onset between January 2015 and December 2019
Number of reporting countries: 29 (AT, BE, CY, CZ, DE, DK, EE, EL, ES, FI, FR, HR, HU, IE, IS, IT, LT, LU, LV, MT, NL, NO, PL, PT, RO, SE, SI, SK and UK)
Number of cases reported: 38,500
Number of countries of infection: 111

↓

Reporting countries that provided case numbers every year and provided country of infection for at least 50% of their cases over the study period
Number of reporting countries: 26 (AT, CY, CZ, DE, DK, EE, EL, ES, FI, FR, HU, IE, IT, LT, LU, LV, MT, NL, NO, PL, PT, RO, SE, SI, SK and UK)
Number of cases reported: 38,197
Number of countries of infection: 109

↓

Cases with known country of infections
Excluded cases with unknown country of infection and cases with multiple place of infection
Number of reporting countries: 26 (AT, CY, CZ, DE, DK, EE, EL, ES, FI, FR, HU, IE, IT, LT, LU, LV, MT, NL, NO, PL, PT, RO, SE, SI, SK and UK)
Number of cases reported: 36,291
Number of countries of infection: 109

↓

Cases infected in Africa
Number of reporting countries: 26 (AT, CY, CZ, DE, DK, EE, EL, ES, FI, FR, HU, IE, IT, LT, LU, LV, MT, NL, NO, PL, PT, RO, SE, SI, SK and UK)
Number of cases reported: 34,238
Number of countries of infection: 53

↓

Countries of infections that were associated to at least two cases, of which one or more is a confirmed case, and that were either reported by two different reporting countries or reported over multiple years
Number of reporting countries: 26 (AT, CY, CZ, DE, DK, EE, EL, ES, FI, FR, HU, IE, IT, LT, LU, LV, MT, NL, NO, PL, PT, RO, SE, SI, SK and UK)
Number of cases reported: 34,235
Number of countries of infection: 50

Note: AT (Austria), BE (Belgium), BG (Bulgaria), CY (Cyprus), CZ (Czechia), DE (Germany), DK (Denmark), EE (Estonia), EL (Greece), ES (Spain), FI (Finland), FR (France), HR (Croatia), HU (Hungary), IE (Ireland), IS (Iceland), IT (Italy), LT (Lithuania), LV (Latvia), MT (Malta), NL (The Netherlands), NO (Norway), PL (Poland), PT (Portugal), RO (Romania), SE (Sweden), SI (Slovenia), SK (Slovakia) and UK (The United Kingdom)

Dengue

Probable and confirmed travel-related cases with (estimated) date of onset between January 2015 and December 2019
Number of reporting countries: 24 (AT, DE, EE, EL, ES, FI, FR, HR, HU, IE, IS, IT, LT, LU, LV, MT, NO, PL, PT, RO, SE, SI, SK and UK)
Number of cases reported: 12,856
Number of countries of infection: 139

↓

Reporting countries that provided case numbers every year and provided country of infection for at least 50% of their cases over the study period
Number of reporting countries: 22 (AT, DE, EE, EL, ES, FI, FR, HU, IE, IT, LT, LU, LV, MT, NO, PL, PT, RO, SE, SI, SK and UK)
Number of cases reported: 12,844
Number of countries of infection: 139

↓

Cases with known country of infections
Excluded cases with unknown country of infection and cases with multiple place of infection
Number of reporting countries: 22 (AT, DE, EE, EL, ES, FI, FR, HU, IE, IT, LT, LU, LV, MT, NO, PL, PT, RO, SE, SI, SK and UK)
Number of cases reported: 11,642
Number of countries of infection: 139

↓

Cases infected in Africa
Number of reporting countries: 19 (AT, CY, CZ, DE, DK, EE, EL, ES, FI, FR, HU, IE, IT, LT, LU, LV, MT, NL, NO, PL, PT, RO, SE, SI, SK and UK)
Number of cases reported: 966
Number of countries of infection: 49

↓

Countries of infections that were associated to at least two cases, of which one or more is a confirmed case, and that were either reported by two different reporting countries or reported over multiple years
Number of reporting countries: 19 (AT, CY, CZ, DE, DK, EE, EL, ES, FI, FR, HU, IE, IT, LT, LU, LV, MT, NL, NO, PL, PT, RO, SE, SI, SK and UK)
Number of cases reported: 956
Number of countries of infection: 41

Chikungunya

Probable and confirmed travel-related cases with (estimated) date of onset between January 2015 and December 2019
Number of reporting countries: 22 (AT, BE, CZ, DE, DK, EL, ES, FI, FR, HU, IE, IT, LU, MT, NL, NO, PT, RO, SE, SI, SK and UK)
Number of cases reported: 2,451
Number of countries of infection: 79

↓

Reporting countries that provided case numbers every year and provided country of infection for at least 50% of their cases over the study period
Number of reporting countries: 18 (CZ, DE, EL, ES, FI, FR, HU, IE, IT, LU, LV, MT, PL, PT, RO, SE, SI and UK)
Number of cases reported: 1,801
Number of countries of infection: 77

↓

Cases with known country of infections
Excluded cases with unknown country of infection and cases with multiple place of infection
Number of reporting countries: 18 (CZ, DE, EL, ES, FI, FR, HU, IE, IT, LU, LV, MT, PL, PT, RO, SE, SI and UK)
Number of cases reported: 1,532
Number of countries of infection: 77

↓

Cases infected in Africa
Number of reporting countries: 8 (CZ, DE, ES, FR, HU,IT, SE and UK)
Number of cases reported: 173
Number of countries of infection: 30

↓

Countries of infections that were associated to at least two cases, of which one or more is a confirmed case, and that were either reported by two different reporting countries or reported over multiple years
Number of reporting countries: 8 (CZ, DE, ES, FR, HU,IT, SE and UK)
Number of cases reported: 161
Number of countries of infection: 20

Zika virus disease

Probable and confirmed travel-related cases plus cases with unknown classification stutus, with (estimated) date of onset between January 2015 and December 2019
Number of reporting countries: 22 (AT, BE, CZ, DE, DK, EL, ES, FI, FR, HU, IE, IT, LU, MT, NL, NO, PT, RO, SE, SI, SK and UK)
Number of cases reported: 2,451
Number of countries of infection: 60

↓

Cases with known country of infections
Excluded cases with unknown country of infection and cases with multiple place of infection
Number of reporting countries: 22 (AT, BE, CZ, DE, DK, EL, ES, FI, FR, HU, IE, IT, LU, MT, NL, NO, PT, RO, SE, SI, SK and UK)
Number of cases reported: 2,149
Number of countries of infection: 60

↓

Cases infected in Africa
Number of reporting countries: 6 (DE, ES, FI, FR, NL, PT)
Number of cases reported: 16
Number of countries of infection: 8

West Nile virus infection

Probable and confirmed travel-related cases with (estimated) date of onset between January 2015 and December 2019
Number of reporting countries: 17 (AT, BE, BG, CY, CZ, DE, EL, ES, FR, HR, HU, IT, NL, SE, SI, SK and UK)
Number of cases reported: 113
Number of countries of infection: 28

↓

Cases with known country of infections
Excluded cases with unknown country of infection and cases with multiple place of infection
Number of reporting countries: 16 (AT, BE, BG, CY, CZ, DE, EL, ES, FR, HR, HU, NL, SE, SI, SK and UK)
Number of cases reported: 87
Number of countries of infection: 28

↓

Cases infected in Africa
Number of reporting countries: 4 (BE, FR, SE, UK)
Number of cases reported: 9
Number of countries of infection: 5

Rift Valley fever

Probable and confirmed travel-related cases with (estimated) date of onset between January 2015 and December 2019
Number of reporting countries: 1 (FR)
Number of cases reported: 4
Number of countries of infection: 2

↓

Cases with known country of infections
Excluded cases with unknown country of infection and cases with multiple place of infection
Number of reporting countries: 1 (FR)
Number of cases reported: 4
Number of countries of infection: 2

↓

Cases infected in Africa
Number of reporting countries: 1 (FR)
Number of cases reported: 4
Number of countries of infection: 2

Yellow fever

Probable and confirmed travel-related cases with (estimated) date of onset between January 2015 and December 2019
Number of reporting countries: 5 (CZ, FR, NL, RO and UK)
Number of cases reported: 12
Number of countries of infection: 3

↓

Cases with known country of infections
Excluded cases with unknown country of infection and cases with multiple place of infection
Number of reporting countries: 5 (CZ, FR, NL, RO and UK)
Number of cases reported: 12
Number of countries of infection: 3

↓

Cases infected in Africa
Number of reporting countries: 1 (NL)
Number of cases reported: 1
Number of countries of infection: 1

Supplementary material 2

Number of cases and rates of infection per year for malaria, dengue and chikungunya virus disease per country of infection and region

MALARIA		2015		2016		2017		2018		2019		2015-2019		
	**Country of infection**	**Number of cases**	**Rate of infection (per 100,000 travellers)**	**Number of cases**	**Rate of infection (per 100,000 travellers)**	**Number of cases**	**Rate of infection (per 100,000 travellers)**	**Number of cases**	**Rate of infection (per 100,000 travellers)**	**Number of cases**	**Rate of infection (per 100,000 travellers)**	**Number of cases**	**Rate of infection (per 100,000 travellers)**	**95% Confidence interval**
**Eastern Africa**	Burundi	9	138.3	11	263.4	13	197.5	14	204.0	15	216.4	62	199.6	153.0-255.9
	Comoros	2	19.0	6	54.8	13	82.5	59	280.9	68	337.3	148	188.8	159.6-221.8
	Djibouti	2	9.4	2	8.2	4	14.8	9	31.5	13	45.0	30	23.1	15.6-32.9
	Eritrea	404	1955.9	121	415.3	46	182.0	43	144.5	19	60.6	633	464.7	429.2-502.4
	Ethiopia	83	46.8	70	45.0	47	32.1	49	28.4	32	15.7	281	32.8	29.1-36.9
	Kenya	113	26.4	106	23.2	110	22.0	77	13.4	136	23.3	542	21.3	19.6-23.2
	Madagascar	26	19.2	7	4.8	23	15.6	16	8.8	14	7.4	86	10.7	8.6-13.3
	Malawi	40	163.2	39	153.9	43	174.0	39	145.4	32	107.6	193	147.2	127.2-169.5
	Mauritius	0	0.0	0	0.0	0	0.0	0	0.0	0	0.0	0	0.0	-
	Mayotte (France)	0	0.0	0	0.0	0	0.0	0	0.0	0	0.0	0	0.0	-
	Mozambique	76	114.9	60	90.7	61	90.7	53	66.7	36	40.5	286	77.7	69.0-87.3
	Réunion (France)	0	0.0	0	0.0	0	0.0	0	0.0	0	0.0	0	0.0	-
	Rwanda	18	53.6	32	79.6	34	71.0	15	30.2	20	36.5	119	52.6	43.6-63.0
	Seychelles	0	0.0	0	0.0	0	0.0	0	0.0	0	0.0	0	0.0	-
	Somalia	27	75.9	17	51.8	8	19.3	6	12.0	6	9.8	64	28.9	22.2-36.9
	South Sudan	2	25.2	6	100.3	18	299.7	15	204.5	18	236.4	59	169.1	128.7-218.2
	Tanzania	91	35.2	49	17.8	85	27.6	55	14.8	79	20.6	359	22.5	20.2-24.9
	Uganda	103	86.5	167	140.7	171	126.6	135	85.4	306	203.6	882	129.5	121.1-138.3
	Zambia	15	37.8	18	38.6	27	57.4	18	35.7	31	62.1	109	46.6	38.3-56.2
	Zimbabwe	6	8.2	3	3.5	6	6.2	2	1.9	7	6.1	24	5.1	3.2-7.5
	Total Eastern Africa	*1017*	*37.6*	*714*	*24.7*	*709*	*22.5*	*605*	*17.0*	*832*	*22.7*	*3877*	*24.3*	*23.5-25.1*
**Central Africa**	Angola	200	56.4	233	76.7	110	37.6	107	35.5	102	35.0	752	48.7	45.3-52.3
	Cameroon	721	390.9	759	388.9	844	443.7	867	465.2	865	441.0	4056	425.9	412.9-439.2
	Central African Republic	210	3188.1	150	1603.9	146	1458.8	136	1306.9	148	1159.9	790	1608.5	1498.3-1724.7
	Chad	60	288.3	61	319.6	41	199.0	72	333.6	123	572.4	357	344.7	309.9-382.4
	Congo (Brazzaville)	182	151.6	301	301.2	169	223.1	192	284.0	242	363.7	1086	252.6	237.8-268.1
	Democratic Republic of the Congo	86	140.6	117	190.9	78	121.2	102	157.7	114	157.9	497	153.5	140.3-167.6
	Equatorial Guinea	230	615.9	228	664.3	205	615.0	221	1170.1	230	499.8	1114	655.7	617.7-695.4
	Gabon	59	78.0	95	143.6	79	126.5	126	205.5	129	192.7	488	146.7	134.0-160.4
	São Tomé and Príncipe	5	23.2	3	11.9	0	0.0	2	5.5	1	2.6	11	7.2	3.6-13.0
	Total Central Africa	*1753*	*198.6*	*1947*	*239.2*	*1672*	*214.3*	*1825*	*237.6*	*1954*	*240.8*	*9151*	*225.6*	*221.0-230.3*
**Northern Africa**	Algeria	0	0.0	1	<0.1	0	0.0	0	0.0	2	0.1	3	<0.1	0.0-0.1
	Canary Islands (Spain)	0	0.0	0	0.0	0	0.0	0	0.0	0	0.0	0	0.0	-
	Egypt	2	0.1	0	0.0	0	0.0	1	<0.1	2	0.1	5	0.0	0.0-0.1
	Libya	6	148.8	5	201.2	3	96.9	0	0.0	0	0.0	14	93.1	50.9-156.1
	Madeira (Portugal)	0	0.0	0	0.0	0	0.0	0	0.0	0	0.0	0	0.0	-
	Morocco	1	<0.1	0	0.0	2	<0.1	4	0.1	2	<0.1	9	<0.1	0.0-0.1
	Sudan	129	343.0	73	170.1	47	73.8	74	101.6	94	167.2	417	152.6	138.3-168.0
	Tunisia	0	0.0	0	0.0	0	0.0	0	0.0	0	0.0	0	0.0	-
	Western Sahara	1	6.2	0	0.0	1	3.5	1	3.3	0	0.0	3	2.2	0.4-6.3
	Total Northern Africa	*139*	*1.0*	*79*	*0.6*	*53*	*0.4*	*80*	*0.5*	*100*	*0.6*	*451*	*0.6*	*0.5-0.7*
**Southern Africa**	Botswana	0	0.0	0	0.0	2	11.2	5	28.0	0	0.0	7	7.8	3.1-16.0
	Lesotho	0	0.0	0	0.0	0	0.0	0	0.0	0	0.0	0	0.0	-
	Namibia	1	0.9	4	3.0	1	0.6	4	2.3	3	1.8	13	1.8	0.9-3.0
	South Africa	10	0.7	8	0.5	33	1.8	19	1.1	9	0.5	79	0.9	0.7-1.2
	Swaziland	0	0.0	0	0.0	0	0.0	0	0.0	0	0.0	0	0.0	-
	Total Southern Africa	*11*	*0.7*	*12*	*0.7*	*36*	*1.8*	*28*	*1.4*	*12*	*0.6*	*99*	*1.1*	*0.9-1.3*

**Western Africa**	Benin	84	160.7	67	121.5	126	226.1	123	223.7	134	224.5	534	192.2	176.2-209.2
	Burkina Faso	209	509.3	223	324.9	236	339.0	237	322.9	205	275.6	1110	339.4	319.7-359.9
	Cabo Verde	1	0.2	4	0.8	2	0.3	3	0.5	0	0.0	10	0.3	0.2-0.6
	Côte d'Ivoire	842	453.3	686	342.3	1037	467.8	1046	414.5	939	355.6	4550	404.7	393.1-416.7
	Gambia	116	158.7	83	93.2	23	33.0	33	34.8	27	20.6	282	61.6	54.7-69.3
	Ghana	448	161.2	390	137.0	423	140.5	396	118.9	361	104.0	2018	130.7	125.1-136.5
	Guinea	227	689.1	238	582.2	278	610.2	320	695.9	381	664.5	1444	648.4	615.4-682.7
	Guinea Bissau	34	417.5	34	263.7	21	66.3	26	74.6	35	84.7	150	116.4	98.5-136.6
	Liberia	29	407.2	31	375.3	25	207.5	41	307.6	19	232.5	145	296.3	250.1-348.7
	Mali	380	347.5	334	302.7	278	212.9	328	228.0	269	168.6	1589	243.1	231.3-255.4
	Mauritania	5	13.7	5	13.9	4	10.1	1	2.5	1	2.2	16	8.0	4.6-13.1
	Niger	14	40.5	26	75.7	35	89.8	34	85.6	44	104.7	153	80.7	68.4-94.5
	Nigeria	958	165.9	1079	195.2	1345	248.6	1210	206.0	1230	200.5	5822	202.7	197.6-208.0
	Saint Helena	0	0.0	0	0.0	0	0.0	0	0.0	0	0.0	0	0.0	-
	Senegal	240	56.8	163	35.0	207	40.9	208	38.4	155	26.9	973	38.7	36.3-41.2
	Sierra Leone	122	589.8	202	609.4	236	711.4	248	779.3	263	833.8	1071	712.2	670.2-756.2
	Togo	104	197.1	128	285.6	165	356.7	174	323.1	219	330.5	790	299.3	278.8-320.9
	Total Western Africa	*3813*	*150.6*	*3693*	*137.3*	*4441*	*151.2*	*4428*	*138.5*	*4282*	*125.9*	*20657*	*140.0*	*138.1-141.9*
**Total Africa**		**6733**	**30.6**	**6445**	**31.1**	**6911**	**29.9**	**6966**	**27.0**	**7180**	**26.3**	**34235**	**28.8**	**28.5-29.1**

DENGUE		2015		2016		2017		2018		2019		2015-2019		
	Country of infection	Number of cases	Rate of infection (per 100,000 travellers)	Number of cases	Rate of infection (per 100,000 travellers)	Number of cases	Rate of infection (per 100,000 travellers)	Number of cases	Rate of infection (per 100,000 travellers)	Number of cases	Rate of infection (per 100,000 travellers)	Number of cases	Rate of infection (per 100,000 travellers)	95% Confidence interval
**Eastern Africa**	Burundi	0	0.0	0	0.0	0	0.0	0	0.0	0	0.0	0	0.0	-
	Comoros	1	9.5	0	0.0	0	0.0	0	0.0	8	39.7	9	11.5	5.3-21.8
	Djibouti	1	4.8	2	8.3	0	0.0	2	7.1	5	17.5	10	7.8	3.7-14.3
	Eritrea	2	10.0	1	3.5	3	12.0	0	0.0	0	0.0	6	4.5	1.6-9.8
	Ethiopia	1	0.6	1	0.7	6	4.2	1	0.6	6	3.0	15	1.8	1.0-3.0
	Kenya	3	0.7	12	2.7	22	4.6	10	1.8	31	5.5	78	3.2	2.5-4.0
	Madagascar	1	0.7	1	0.7	0	0.0	4	2.2	2	1.1	8	1.0	0.4-2.0
	Malawi	0	0.0	0	0.0	0	0.0	0	0.0	0	0.0	0	0.0	-
	Mauritius	1	0.2	2	0.3	3	0.5	1	0.1	3	0.4	10	0.3	0.1-0.6
	Mayotte (France)	0	0.0	2	8.4	0	0.0	0	0.0	4	6.7	6	3.1	1.1-6.7
	Mozambique	1	1.5	1	1.5	4	6.0	1	1.3	4	4.5	11	3.0	1.5-5.4
	Réunion (France)	0	0.0	1	0.2	4	0.7	34	5.2	117	18.1	156	5.2	4.4-6.1
	Rwanda	0	0.0	0	0.0	0	0.0	0	0.0	0	0.0	0	0.0	-
	Seychelles	2	1.6	18	11.3	43	21.8	9	4.4	10	4.7	82	9.1	7.2-11.3
	Somalia	4	11.9	3	9.5	8	20.1	5	10.7	15	25.5	35	16.6	11.6-23.1
	South Sudan	0	0.0	0	0.0	0	0.0	0	0.0	0	0.0	0	0.0	-
	Tanzania	5	2.0	6	2.3	7	2.4	16	4.5	34	9.3	68	4.5	3.5-5.7
	Uganda	0	0.0	2	1.8	4	3.1	1	0.7	4	2.8	11	1.7	0.8-3.0
	Zambia	0	0.0	0	0.0	0	0.0	0	0.0	0	0.0	0	0.0	-
	Zimbabwe	0	0.0	0	0.0	0	0.0	0	0.0	0	0.0	0	0.0	-
	Total Eastern Africa	22	0.8	52	1.8	104	3.4	84	2.4	243	6.8	505	3.2	2.9-3.5
**Central Africa**	Angola	2	0.6	4	1.3	3	1.0	16	5.3	3	1.0	28	1.8	1.2-2.6
	Cameroon	4	2.2	3	1.6	6	3.2	6	3.3	6	3.1	25	2.7	1.7-3.9
	Central African Republic	0	0.0	1	10.7	0	0.0	0	0.0	2	15.8	3	6.1	1.3-17.9
	Chad	0	0.0	0	0.0	0	0.0	0	0.0	0	0.0	0	0.0	-
	Congo (Brazzaville)	2	1.7	3	3.0	2	2.6	2	3.0	1	1.5	10	2.3	1.1-4.3
	Democratic Republic of the Congo	0	0.0	5	8.2	0	0.0	3	4.7	7	9.8	15	4.7	2.6-7.7
	Equatorial Guinea	4	10.7	1	2.9	0	0.0	1	5.3	0	0.0	6	3.5	1.3-7.7
	Gabon	0	0.0	2	3.0	1	1.6	0	0.0	2	3.0	5	1.5	0.5-3.5
	São Tomé and Príncipe	0	0.0	0	0.0	0	0.0	0	0.0	0	0.0	0	0.0	-
	Total Central Africa	*12*	*1.4*	*19*	*2.3*	*12*	*1.5*	*28*	*3.7*	*21*	*2.6*	*92*	*2.3*	*1.8-2.8*
**Northern Africa**	Algeria	0	0.0	0	0.0	0	0.0	0	0.0	0	0.0	0	0.0	-
	Canary Islands (Spain)	0	0.0	0	0.0	0	0.0	0	0.0	0	0.0	0	0.0	-
	Egypt	1	<0.1	0	0.0	12	0.5	4	0.1	6	0.2	23	0.2	0.1-0.3
	Libya	0	0.0	0	0.0	0	0.0	0	0.0	0	0.0	0	0.0	-
	Madeira (Portugal)	0	0.0	0	0.0	0	0.0	0	0.0	0	0.0	0	0.0	-
	Morocco	1	<0.1	0	0.0	0	0.0	0	0.0	1	<0.1	2	<0.1	0.00-0.03*
	Sudan	1	2.7	0	0.0	1	1.6	1	1.4	2	3.6	5	1.9	0.6-4.4
	Tunisia	0	0.0	0	0.0	0	0.0	0	0.0	0	0.0	0	0.0	-
	Western Sahara	0	0.0	0	0.0	0	0.0	0	0.0	0	0.0	0	0.0	-
	Total Northern Africa	*3*	*<0.1*	*0*	*0.0*	*13*	*0.1*	*5*	*<0.1*	*9*	*0.1*	*30*	*<0.1*	*0.0-0.1*
**Southern Africa**	Botswana	1	6.8	0	0.0	0	0.0	1	5.8	1	4.3	3	3.4	0.7-10.0
	Lesotho	0	0.0	0	0.0	1	164.7	0	0.0	2	325.2	3	107.9	22.2-315-3
	Namibia	1	0.9	2	1.5	0	0.0	1	0.6	1	0.6	5	0.7	0.2-1.6
	South Africa	0	0.0	3	0.2	2	0.1	0	0.0	2	0.1	7	0.1	0.0-0.2
	Swaziland	0	0.0	0	0.0	0	0.0	0	0.0	0	0.0	0	0.0	-
	Total Southern Africa	*2*	*0.1*	*5*	*0.3*	*3*	*0.2*	*2*	*0.1*	*6*	*0.3*	*18*	*0.2*	*0.1-0.3*

**Western Africa**	Benin	4	7.7	5	9.1	3	5.4	1	1.8	3	5.0	16	5.8	3.3-9.4
	Burkina Faso	0	0.0	17	25.1	13	18.8	2	2.7	16	21.6	48	14.8	10.9-19.6
	Cabo Verde	1	0.2	0	0.0	0	0.0	1	0.2	0	0.0	2	0.1	0.0-0.3
	Côte d'Ivoire	2	1.1	0	0.0	55	24.9	5	2.0	49	18.6	111	9.9	8.2-11.9
	Gambia	0	0.0	0	0.0	0	0.0	0	0.0	0	0.0	0	0.0	-
	Ghana	6	2.2	1	0.4	9	3.1	1	0.3	3	0.9	20	1.3	0.8-2.0
	Guinea	0	0.0	0	0.0	0	0.0	2	4.4	2	3.5	4	1.8	0.5-4.6
	Guinea Bissau	0	0.0	0	0.0	0	0.0	0	0.0	0	0.0	0	0.0	-
	Liberia	0	0.0	0	0.0	0	0.0	0	0.0	0	0.0	0	0.0	-
	Mali	1	0.9	1	0.9	4	3.1	2	1.4	6	3.8	14	2.2	1.2-3.6
	Mauritania	4	11.0	0	0.0	0	0.0	2	4.9	0	0.0	6	3.0	1.1-6.6
	Niger	0	0.0	2	5.8	0	0.0	1	2.5	1	2.4	4	2.1	0.6-5.4
	Nigeria	2	0.3	9	1.6	15	2.8	6	1.0	8	1.3	40	1.4	1.0-1.9
	Saint Helena	0	0.0	0	0.0	0	0.0	0	0.0	0	0.0	0	0.0	-
	Senegal	8	1.9	7	1.5	3	0.6	2	0.4	3	0.5	23	0.9	0.6-1.4
	Sierra Leone	0	0.0	3	9.3	0	0.0	0	0.0	0	0.0	3	2.0	0.4-5.9
	Togo	3	5.7	7	15.8	4	8.7	2	3.7	4	6.1	20	7.6	4.7-11.8
	Total Western Africa	*31*	*1.2*	*52*	*2.0*	*106*	*3.6*	*27*	*0.9*	*95*	*2.8*	*311*	*2.1*	*1.9-2.4*
**Total Africa**		**70**	**0.3**	**128**	**0.6**	**239**	**1.0**	**146**	**0.6**	**374**	**1.4**	**956**	**0.8**	**0.8-0.9**

* result provided at two decimals for clarity

CHIKUNGUNYA		2015		2016		2017		2018		2019		2015-2019		
	**Country of infection**	**Number of cases**	**Rate of infection (per 100,000 travellers)**	**Number of cases**	**Rate of infection (per 100,000 travellers)**	**Rate of infection (per 100,000 travellers)**	**Rate of infection**	**Rate of infection (per 100,000 travellers)**	**Rate of infection**	**Rate of infection (per 100,000 travellers)**	**Rate of infection**	**Number of cases**	**Rate of infection (per 100,000 travellers)**	**95% Confidence interval**
	Burundi	0	0.0	0	0.0	0	0.0	0	0.0	0	0.0	0	0.0	-
	Comoros	0	0.0	0	0.0	0	0.0	0	0.0	0	0.0	0	0.0	-
	Djibouti	0	0.0	0	0.0	1	3.8	0	0.0	4	14.2	5	3.9	1.3-9.2
	Eritrea	0	0.0	0	0.0	0	0.0	2	7.6	1	3.5	3	2.4	0.5-7.1
	Ethiopia	0	0.0	0	0.0	0	0.0	0	0.0	5	2.7	5	0.6	0.2-1.5
	Kenya	0	0.0	2	0.5	1	0.2	27	5.1	4	0.7	34	1.4	1.0-2.0
	Madagascar	0	0.0	0	0.0	0	0.0	0	0.0	0	0.0	0	0.0	-
	Malawi	0	0.0	0	0.0	0	0.0	0	0.0	0	0.0	0	0.0	-
	Mauritius	0	0.0	0	0.0	0	0.0	0	0.0	0	0.0	0	0.0	-
	Mayotte (France)	0	0.0	0	0.0	0	0.0	0	0.0	0	0.0	0	0.0	-
**Eastern Africa**	Mozambique	0	0.0	0	0.0	0	0.0	0	0.0	0	0.0	0	0.0	-
	Réunion (France)	0	0.0	0	0.0	0	0.0	0	0.0	0	0.0	0	0.0	-
	Rwanda	0	0.0	0	0.0	0	0.0	0	0.0	0	0.0	0	0.0	-
	Seychelles	0	0.0	0	0.0	0	0.0	0	0.0	0	0.0	0	0.0	-
	Somalia	0	0.0	19	65.0	3	8.3	2	4.7	5	9.2	29	15.0	10.0-21.5
	South Sudan	0	0.0	0	0.0	0	0.0	0	0.0	0	0.0	0	0.0	-
	Tanzania	1	0.4	0	0.0	0	0.0	4	1.2	1	0.3	6	0.4	0.2-0.9
	Uganda	0	0.0	1	0.9	1	0.8	0	0.0	0	0.0	2	0.3	0.0-1.2
	Zambia	0	0.0	0	0.0	0	0.0	0	0.0	0	0.0	0	0.0	-
	Zimbabwe	0	0.0	0	0.0	0	0.0	0	0.0	0	0.0	0	0.0	-
	Total Eastern Africa	1	0.0	22	0.8	6	0.2	35	1.0	20	0.6	84	0.6	0.4-0.7
	Angola	0	0.0	0	0.0	0	0.0	0	0.0	0	0.0	0	0.0	-
	Cameroon	2	1.1	2	1.0	2	1.1	1	0.5	0	0.0	7	0.7	0.3-1.5
	Central African Republic	0	0.0	0	0.0	0	0.0	0	0.0	0	0.0	0	0.0	-
	Chad	0	0.0	0	0.0	0	0.0	0	0.0	0	0.0	0	0.0	-
**Central Africa**	Congo (Brazzaville)	1	0.8	0	0.0	1	1.3	0	0.0	15	22.6	17	4.0	2.3-6.3
	Democratic Republic of the Congo	1	1.7	0	0.0	0	0.0	0	0.0	11	15.6	12	3.8	1.9-6.6
	Equatorial Guinea	3	8.0	3	8.8	3	9.0	0	0.0	1	2.2	10	5.9	2.8-10.9
	Gabon	0	0.0	0	0.0	0	0.0	0	0.0	2	3.0	2	0.6	0.1-2.2
	São Tomé and Príncipe	0	0.0	0	0.0	0	0.0	0	0.0	0	0.0	0	0.0	-
	Total Central Africa	*7*	*0.8*	*5*	*0.6*	*6*	*0.8*	*1*	*0.1*	*29*	*3.6*	*48*	*1.2*	0.9-1.6
	Algeria	0	0.0	0	0.0	0	0.0	0	0.0	0	0.0	0	0.0	-
	Canary Islands (Spain)	0	0.0	0	0.0	0	0.0	0	0.0	0	0.0	0	0.0	-
	Egypt	0	0.0	0	0.0	0	0.0	0	0.0	0	0.0	0	0.0	-
	Libya	0	0.0	0	0.0	0	0.0	0	0.0	0	0.0	0	0.0	-
**Northern Africa**	Madeira (Portugal)	0	0.0	0	0.0	0	0.0	0	0.0	0	0.0	0	0.0	-
	Morocco	0	0.0	0	0.0	0	0.0	0	0.0	0	0.0	0	0.0	-
	Sudan	0	0.0	1	2.5	0	0.0	2	3.0	0	0.0	3	1.2	0.2-3.5
	Tunisia	0	0.0	0	0.0	0	0.0	0	0.0	0	0.0	0	0.0	-
	Western Sahara	0	0.0	0	0.0	0	0.0	0	0.0	0	0.0	0	0.0	-
	Total Northern Africa	*0*	*0.0*	*1*	*0.0*	*0*	*0.0*	*2*	*0.0*	*0*	*0.0*	*3*	*0.0*	0.00-0.01*
	Botswana	0	0.0	0	0.0	0	0.0	0	0.0	0	0.0	0	0.0	-
	Lesotho	0	0.0	0	0.0	0	0.0	0	0.0	0	0.0	0	0.0	-
**Southern Africa**	Namibia	0	0.0	0	0.0	0	0.0	0	0.0	0	0.0	0	0.0	-
	South Africa	0	0.0	0	0.0	0	0.0	0	0.0	0	0.0	0	0.0	-
	Swaziland	0	0.0	0	0.0	0	0.0	0	0.0	0	0.0	0	0.0	-
	Total Southern Africa	*0*	*0.0*	*0*	*0.0*	*0*	*0.0*	*0*	*0.0*	*0*	*0.0*	*0*	*0.0*	-

**Western Africa**	Benin	0	0.0	0	0.0	0	0.0	0	0.0	0	0.0	0	0.0	-
	Burkina Faso	1	2.5	1	1.5	0	0.0	0	0.0	0	0.0	2	0.6	0.1-2.3
	Cabo Verde	0	0.0	0	0.0	0	0.0	0	0.0	0	0.0	0	0.0	-
	Côte d'Ivoire	1	0.5	1	0.5	2	0.9	0	0.0	1	0.4	5	0.4	0.1-1.0
	Gambia	1	1.5	1	1.2	0	0.0	0	0.0	1	0.8	3	0.7	0.1-2.0
	Ghana	0	0.0	1	0.4	0	0.0	0	0.0	3	0.9	4	0.3	0.1-0.7
	Guinea	0	0.0	0	0.0	0	0.0	0	0.0	0	0.0	0	0.0	-
	Guinea Bissau	0	0.0	0	0.0	0	0.0	0	0.0	0	0.0	0	0.0	-
	Liberia	0	0.0	0	0.0	0	0.0	0	0.0	0	0.0	0	0.0	-
	Mali	1	0.9	0	0.0	0	0.0	0	0.0	1	0.6	2	0.3	0.0-1.1
	Mauritania	0	0.0	0	0.0	0	0.0	0	0.0	0	0.0	0	0.0	-
	Niger	0	0.0	0	0.0	0	0.0	0	0.0	0	0.0	0	0.0	-
	Nigeria	1	0.2	2	0.4	1	0.2	1	0.2	0	0.0	5	0.2	0.1-0.4
	Saint Helena	0	0.0	0	0.0	0	0.0	0	0.0	0	0.0	0	0.0	-
	Senegal	0	0.0	1	0.2	1	0.2	1	0.2	2	0.4	5	0.2	0.1-0.5
	Sierra Leone	0	0.0	0	0.0	0	0.0	0	0.0	0	0.0	0	0.0	-
	Togo	0	0.0	0	0.0	0	0.0	0	0.0	0	0.0	0	0.0	-
	Total Western Africa	*5*	*0.2*	*7*	*0.3*	*4*	*0.1*	*2*	*0.1*	*8*	*0.2*	*26*	*0.2*	0.1-0.3
**Total Africa**		**13**	**0.1**	**35**	**0.2**	**16**	**0.1**	**40**	**0.2**	**57**	**0.2**	**161**	**0.1**	**0.1-0.2**

* result provided at two decimals for clarity

Supplementary material 3

Proportions and numbers of malaria cases in travellers arriving in Europe from Africa, per *Plasmodium* species, year and region, 2015-2019

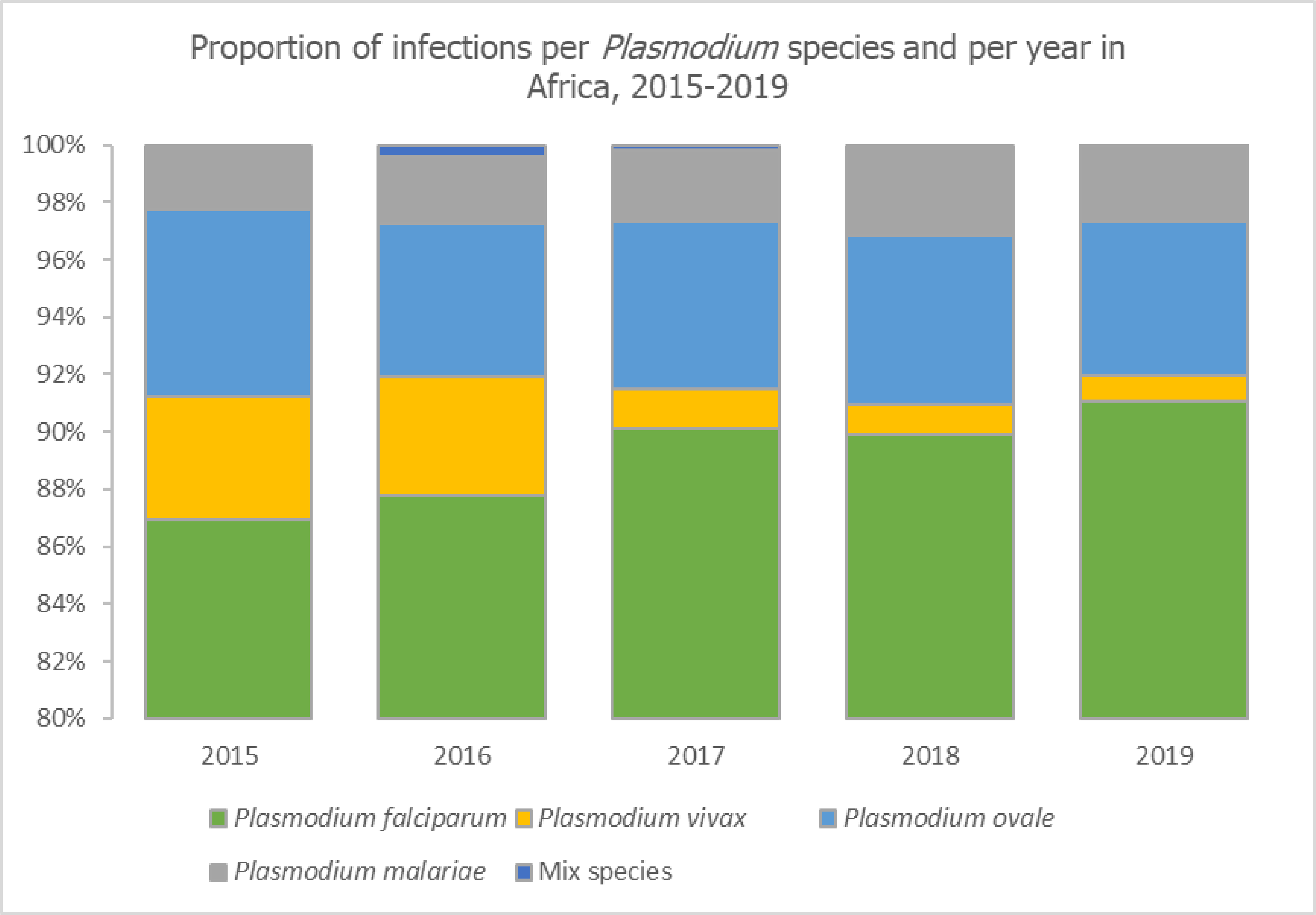
Proportion of infections per Plasmodium species and per year in Africa, 2015-2019
100%
98%
96%
94%
92%
90%
88%
86%
84%
82%
80%
2015
2016
2017
2018
2019
Plasmodium falciparum
Plasmodium vivax
Plasmodium ovale
Plasmodium malariae
Mix species

Number of infections per *Plasmodium* species and per year in Africa, 2015-2019

**Year**	***Plasmodium falciparum***	***Plasmodium vivax***	***Plasmodium ovale***	***Plasmodium malariae***	**Mix species**
2015	5,015	249	374	125	6
2016	5,315	251	325	141	23
2017	5,832	91	378	159	13
2018	5,768	68	376	196	8
2019	6,140	60	364	177	2
**Total**	**28,070**	**719**	**1,817**	**798**	**52**

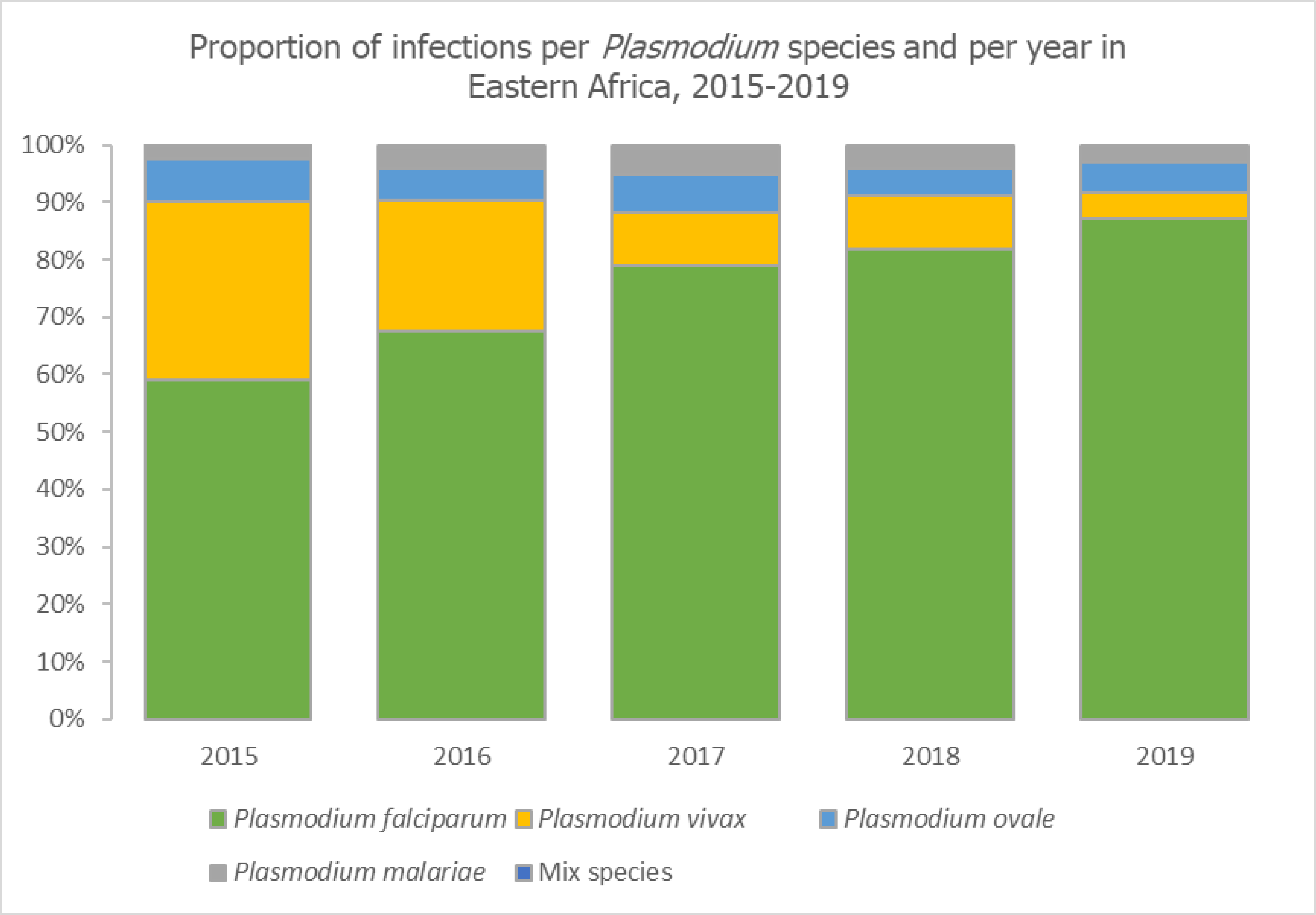
Proportion of infections per *Plasmodium* species and per year in Eastern Africa, 2015-2019
100%
90%
80%
70%
60%
50%
40%
30%
20%
10%
0%
2015
2016
2017
2018
2019
Plasmodium falciparum
Plasmodium vivax
Plasmodium ovale
Plasmodium malariae
Mix species

Proportion of infections per *Plasmodium* species and per year in Eastern Africa, 2015-2019

Year	*Plasmodium falciparum*	*Plasmodium vivax*	*Plasmodium ovale*	*Plasmodium malariae*	Mix species
2015	377	198	48	15	1
2016	424	143	35	24	2
2017	486	56	41	29	3
2018	372	42	21	19	0
2019	657	33	40	23	0
**Total**	**2,316**	**472**	**185**	**110**	**6**

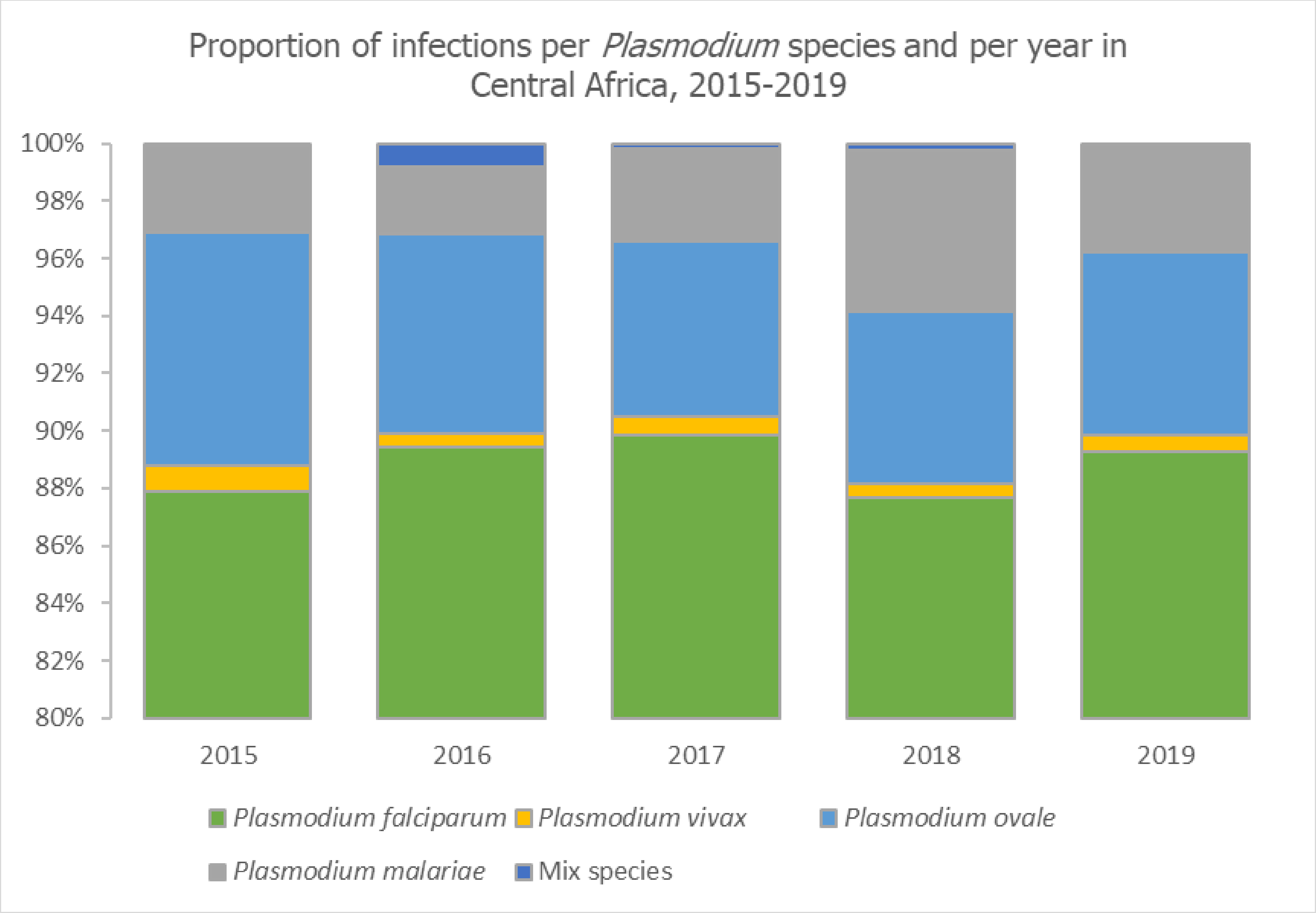

Proportion of infections per *Plasmodium* species and per year in Central Africa, 2015-2019
100%
98%
96%
94%
92%
90%
88%
86%
84%
82%
80%
2015
2016
2017
2018
2019
Plasmodium falciparum
Plasmodium vivax
Plasmodium ovale
Plasmodium malariae
Mix species

Proportion of infections per *Plasmodium* species and per year in Central Africa, 2015-2019

**Year**	***Plasmodium falciparum***	***Plasmodium vivax***	***Plasmodium ovale***	***Plasmodium malariae***	**Mix species**
2015	1,408	14	130	49	1
2016	1,640	9	127	43	15
2017	1,417	10	96	51	3
2018	1,510	8	103	97	4
2019	1,642	11	117	70	0
**Total**	**7,617**	**52**	**573**	**310**	**23**

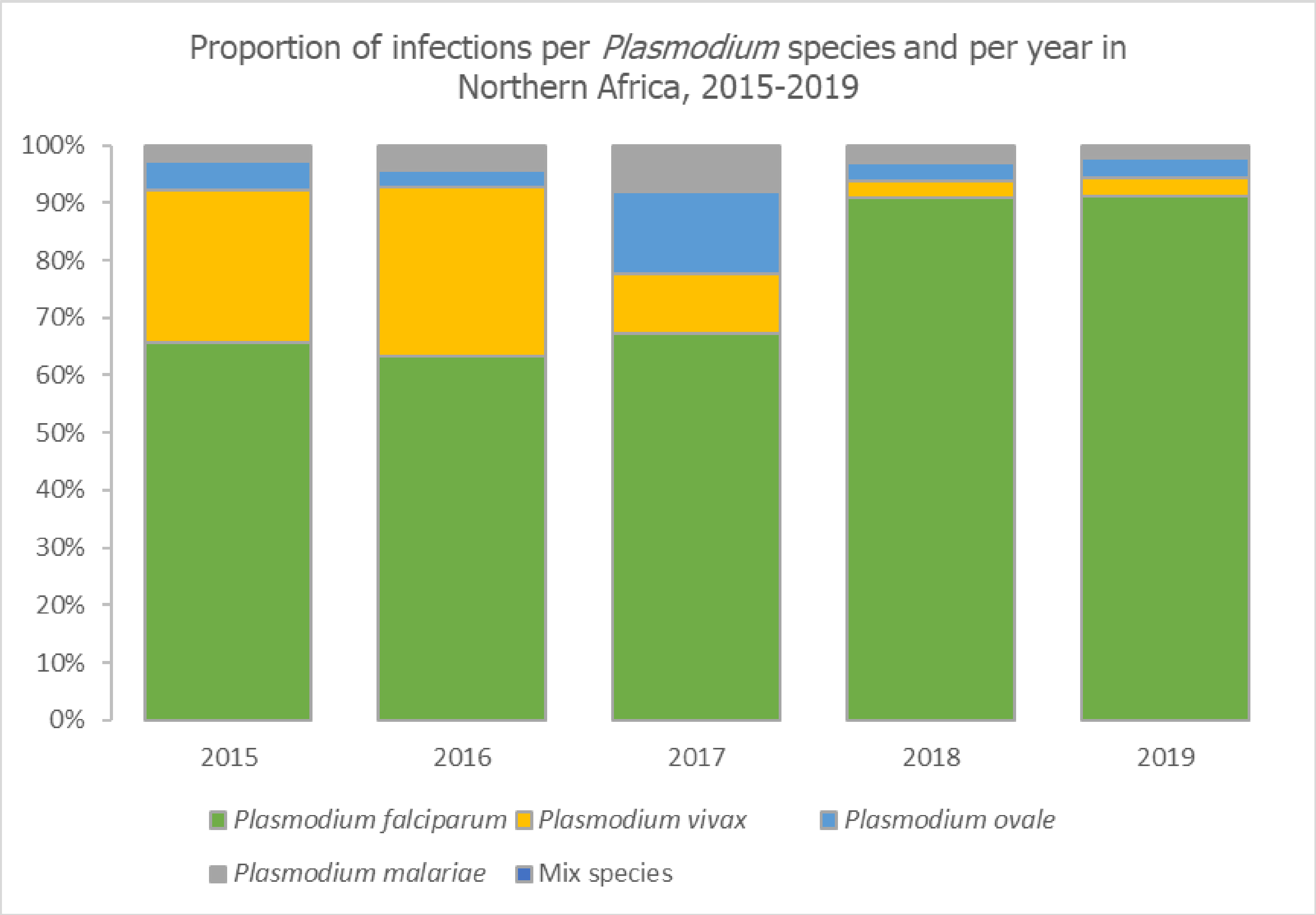
Proportion of infections per *Plasmodium* species and per year in Northern Africa, 2015-2019
100%
90%
80%
70%
60%
50%
40%
30%
20%
10%
0%
2015
2016
2017
2018
2019
Plasmodium falciparum
Plasmodium vivax
Plasmodium ovale
Plasmodium malariae
Mix species

Proportion of infections per *Plasmodium* species and per year in Northern Africa, 2015-2019

**Year**	***Plasmodium falciparum***	***Plasmodium vivax***	***Plasmodium ovale***	***Plasmodium malariae***	**Mix species**
2015	50	20	4	2	0
2016	43	20	2	3	0
2017	33	5	7	4	0
2018	59	2	2	2	0
2019	82	3	3	2	0
**Total**	**267**	**50**	**18**	**13**	**0**

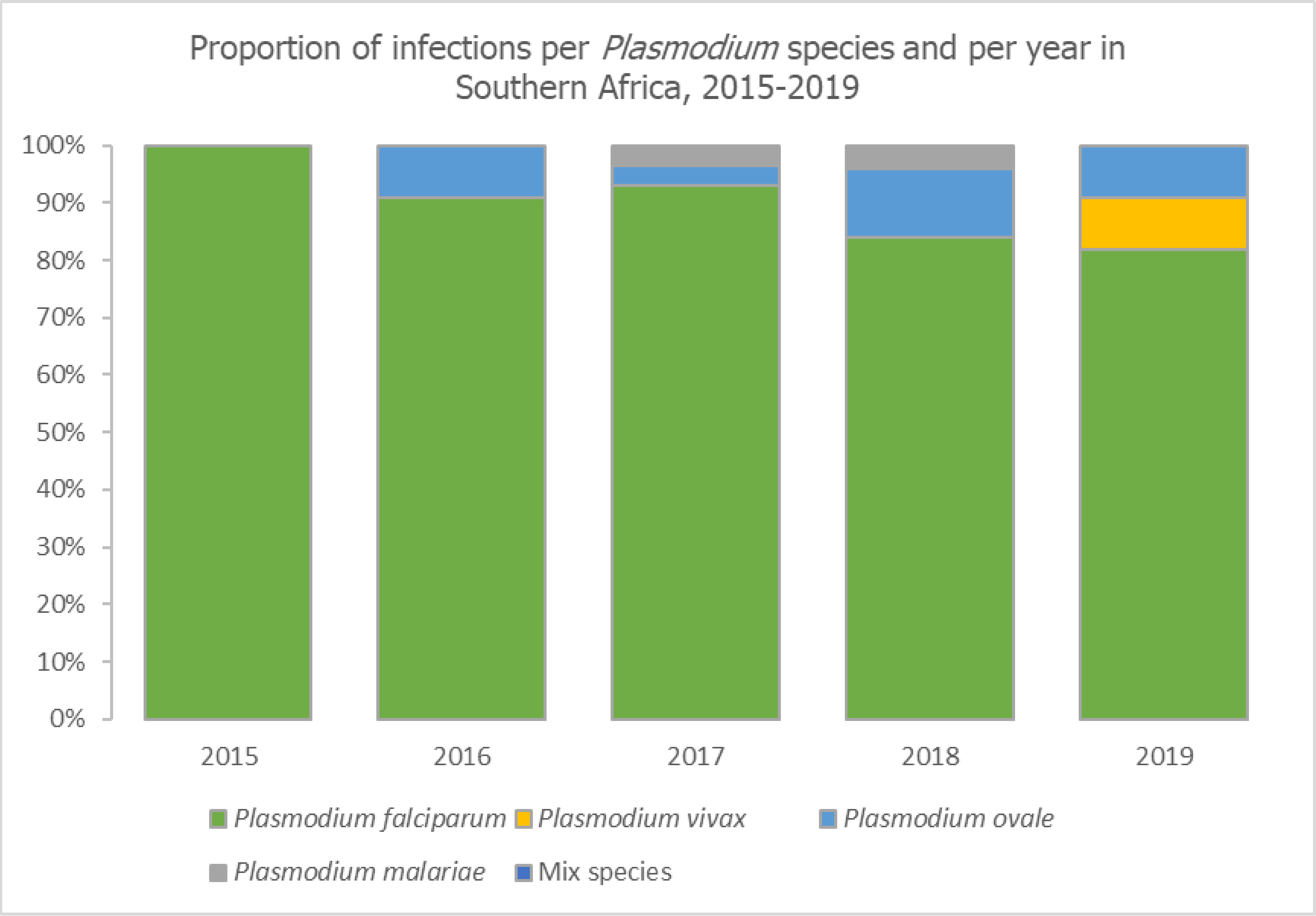
Proportion of infections per Plasmodium species and per year in Southern Africa, 2015-2019
100%
90%
80%
70%
60%
50%
40%
30%
20%
10%
0%
2015
2016
2017
2018
2019
Plasmodium falciparum
Plasmodium vivax
Plasmodium ovale
Plasmodium malariae
Mix species

Proportion of infections per *Plasmodium* species and per year in Southern Africa, 2015-2019

**Year**	***Plasmodium falciparum***	***Plasmodium vivax***	***Plasmodium ovale***	***Plasmodium malariae***	**Mix species**
2015	7	0	0	0	0
2016	10	0	1	0	0
2017	27	0	1	1	0
2018	21	0	3	1	0
2019	9	1	1	0	0
**Total**	**74**	**1**	**6**	**2**	**0**

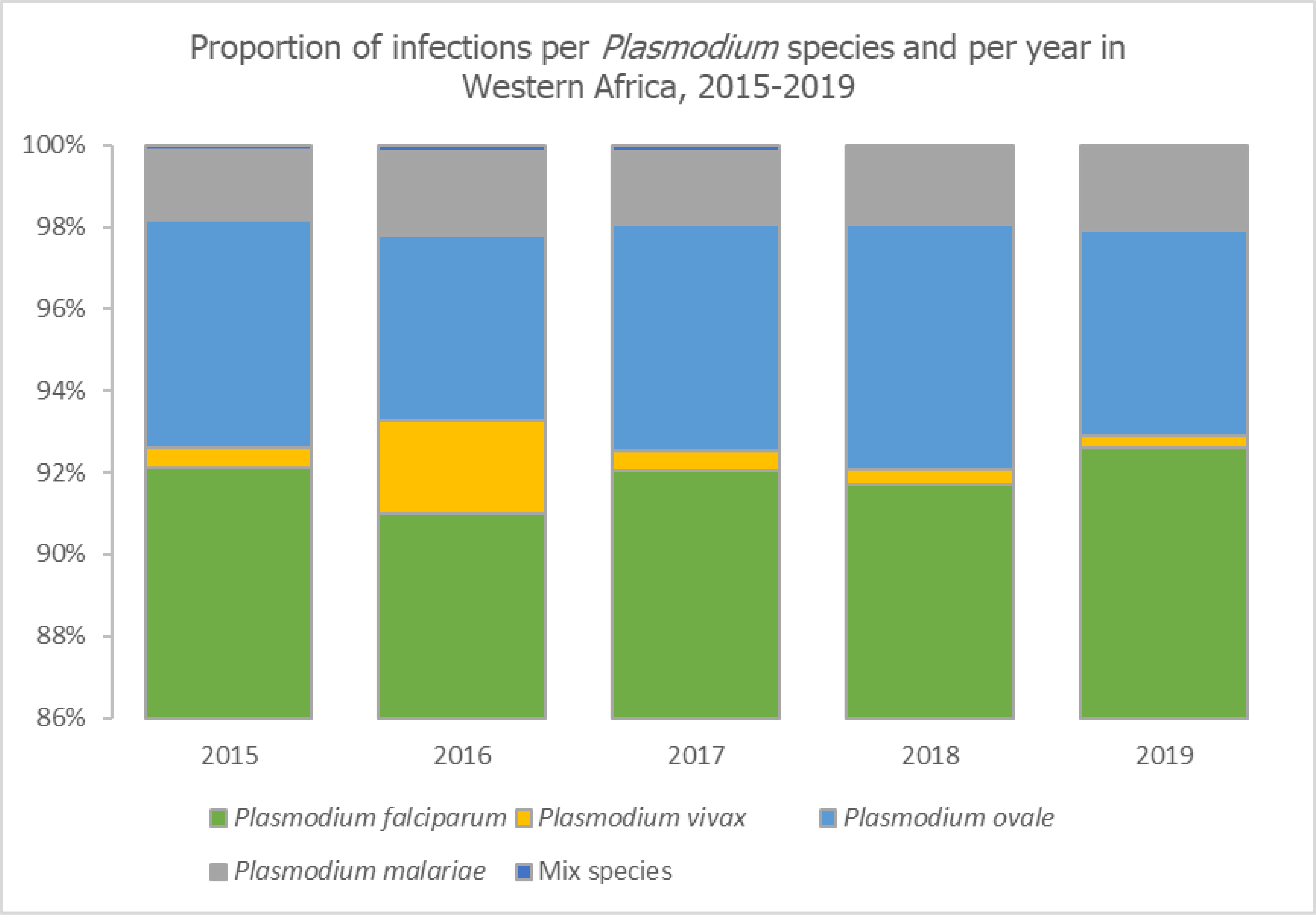
Proportion of infections per Plasmodium species and per year in Western Africa, 2015-2019
100%
98%
96%
94%
92%
90%
88%
86%
2015
2016
2017
2018
2019
Plasmodium falciparum
Plasmodium vivax
Plasmodium ovale
Plasmodium malariae
Mix species

Proportion of infections per *Plasmodium* species and per year in Western Africa, 2015-2019

**Year**	***Plasmodium falciparum***	***Plasmodium vivax***	***Plasmodium ovale***	***Plasmodium malariae***	**Mix species**
2015	3,173	17	192	59	4
2016	3,198	79	160	71	6
2017	3,869	20	233	74	7
2018	3,806	16	247	77	4
2019	3,750,	12	203	82	2
**Total**	**17,796**	**144**	**1,035**	**363**	**23**
